# Loneliness, Aloneness, and Adherence to the Mediterranean Diet in Southern Italian Individuals

**DOI:** 10.3390/nu18030387

**Published:** 2026-01-24

**Authors:** Justyna Godos, Giuseppe Caruso, Marco Antonio Olvera-Moreira, Francesca Giampieri, Kilian Tutusaus, Melannie Toral-Noristz, Raynier Zambrano-Villacres, Alice Leonardi, Rosa M. G. Balzano, Fabio Galvano, Sabrina Castellano, Giuseppe Grosso

**Affiliations:** 1 Department of Biomedical and Biotechnological Sciences, University of Catania, 95123 Catania, Italy; justyna.godos@unict.it (J.G.); giuseppe.grosso@unict.it (G.G.); 2 Departmental Faculty of Medicine, UniCamillus-Saint Camillus International University of Health Sciences, 00131 Rome, Italy; 3 IRCCS San Camillo Hospital, 30126 Venice, Italy; 4 Carrera de Medicina, Universidad Católica de Santiago de Guayaquil, Av. Pdte. Carlos Julio Arosemena Tola, Guayaquil 090615, Ecuador; 5 Department of Clinical Sciences, Università Politecnica delle Marche, 60131 Ancona, Italy; 6 Research Group on Food, Nutritional Biochemistry and Health, Universidad Europea del Atlántico, Isabel Torres 21, 39011 Santander, Spain; 7 Joint Laboratory on Food Science, Nutrition, and Intelligent Processing of Foods, Polytechnic University of Marche, Italy, Universidad Europea del Atlántico, Spain and Jiangsu University, China at Polytechnic University of Marche, 60130 Ancona, Italy; 8 Universidad de La Romana, La Romana 22000, Dominican Republic; 9 Universidade Internacional do Cuanza, Cuito EN250, Bié, Angola; 10 Escuela de Medicina, Universidad Espíritu Santo, Samborondón 0901952, Ecuador; 11 Dirección de Investigación, Universidad ECOTEC, Km.13.5 Samborondón, Samborondón 092302, Ecuador; 12 Department of Educational Sciences, University of Catania, 95124 Catania, Italy

**Keywords:** loneliness, aloneness, Mediterranean diet, diet quality, cognitive function, neurological behavior

## Abstract

**Background/Objectives**: Research across multiple disciplines has explored how nutrition is shaped by social isolation and feelings of loneliness, especially in the elderly population. Evidence from neuroscience highlights that loneliness may alter eating patterns, encouraging emotional eating or other compensatory food behaviors. Conversely, isolation from social contexts is often linked to a reduced variety of nutrient intake. This study set out to examine how psychosocial aspects, particularly social connectedness and feeling alone, relate to adherence to the Mediterranean diet among older adults residing in Sicily, southern Italy. **Methods**: Dietary habits of 883 adults were collected through food frequency questionnaires and assessed for adherence to the Mediterranean diet. Loneliness was measured through a targeted question from a standardized tool designed to capture depressive symptoms. Direct questions asked whether participants were engaged in social networks, such as family, friends and neighborhoods, or religious communities, in order to assess objective aloneness. Logistic regression analyses were performed to assess associations between variables of interest. **Results**: After accounting for potential confounders, both loneliness and aloneness showed an association with stronger adherence to the Mediterranean diet. Specifically, individuals experiencing loneliness and aloneness were less likely to have high adherence to the Mediterranean diet (OR = 0.28, 95% CI: 0.15, 0.51, and OR = 0.26, 95% CI: 0.12, 0.54, respectively). **Conclusions**: These findings underscore the importance of fostering social engagement among older populations, who may particularly benefit from maintaining active social ties to support healthier eating behaviors.

## 1. Introduction

The global population is undergoing a profound demographic shift, with the proportion of older adults steadily increasing across many countries [1]. According to the World Health Organization (WHO), by 2050, the number of people aged 60 years or older will outnumber children under the age of 5, highlighting the significant impact of population aging on healthcare systems and society at large [2]. This demographic transition poses both opportunities and challenges, as increased longevity often brings with it a greater burden of chronic diseases, such as cardiovascular conditions, diabetes, neurodegenerative disorders, and musculoskeletal diseases [3]. Maintaining physical and mental good health in older adults is therefore critical not only to enhancing quality of life but also to mitigating the risk of disability, dependence, and premature mortality [4,5].

Loneliness and aloneness are complex and multidimensional experiences that have received increasing attention in recent years, particularly with regard to their impact on physical and mental health [6]. Despite being often used interchangeably, loneliness and aloneness are distinct constructs that have been differentiated by various authors [7]. Loneliness is defined as the subjective feeling of dissatisfaction with one’s social relationships and a lack of companionship and belonging [8]. On the other hand, aloneness refers to the objective state of being physically solitary or isolated [9]. Hence, while the latter represent an actual situational condition, the other may imply a psychological involvement, including the presence of depressive symptoms or prodromic conditions, such as cognitive decline [10,11,12]. Loneliness and aloneness are important public health topics because of their potential impact on the general population’s health, as widely documented in numerous studies [13]. Loneliness has been associated with an increased risk of mortality [14], cardiovascular disease [15], depression [16], and cognitive decline [17]. Also, loneliness has been linked to poor sleep quality [18] and decreased physical activity levels [19].

The prevalence of loneliness varies across different regions of the world [20]. Recent estimates indicate that approximately one-third of individuals in industrialized nations experience loneliness, with about one in twelve reporting it at a problematic intensity [21]. Studies show that loneliness prevalence might differ between geographical regions (although comparative data are mainly available for Europe), with a clear geographical gradient emerging: northern European countries consistently report lower levels of loneliness across all age groups, while Eastern European countries show considerably higher rates, particularly among older adults [22]. Although often misclassified, the negative impacts associated with these conditions may depend on different processes that need to be differentiated in order to better address the problem and, consequently, apply a more suitable solution.

Research has often investigated the relation between psychosocial factors, such as loneliness and aloneness, with diet quality. Current evidence suggests that individuals who experience loneliness and aloneness tend to engage in unhealthy eating behaviors, such as overeating and poor dietary choices [23,24]. In contrast, a Mediterranean-style diet, which emphasizes plant-based foods, healthy fats, and moderate protein intake, along with the underlying importance of cooking and conviviality [25,26], has been particularly noted for its role in promoting resilience in older adults by supporting both physical and cognitive health [27]. Diet may provide the essential nutrients required for maintaining bodily functions, supporting immune health, and preventing or managing chronic diseases [28]. In addition to its physiological benefits, dietary choices also influence psychological resilience. The consumption of nutrient-rich foods has been linked to improved mood, cognitive function, and overall mental well-being [29,30]. Consistently, there is evidence that unhealthy dietary patterns might be associated with higher risk of depression [31,32] as well as detrimental cognitive outcomes and cognitive decline in the older population [33,34]. This is particularly significant in aging populations, where depression and sleep disturbances can compound the effects of chronic disease and frailty as well as alter neurological behaviors and be prodromic symptoms of more impairing conditions, such as cognitive decline and dementia [5].

Aside from a direct biological effect toward the neurophysiology of the brain counteracting age-related inflammation [35,36,37,38], social eating habits, which often accompany traditional diets like the Mediterranean model, may also play a role on psychosocial aspects, fostering emotional resilience by promoting social interaction and mental engagement, further reducing the risk of isolation and its negative impact on health [39,40]. Aloneness and isolation appear to negatively affect diet quality, as being widowed, living alone, or lacking neighbors are associated with poorer nutrition [41,42]. This may reflect both the absence of social influence in maintaining healthy habits and a reduced motivation to buy and prepare food when it cannot be shared, further highlighting the detrimental impact of isolation on health [43]. Given the relevance of this important topic for public health, the aim of this study was to investigate whether loneliness and aloneness were associated with adherence to the Mediterranean diet, considered as a proxy of a healthy diet in older adults.

## 2. Materials and Methods

### 2.1. Study Design and Population

The analyses for this study were based on a cross-sectional survey including a representative sample of men and women aged 18 years or older as described elsewhere [44]. Participants were randomly selected and enrolled during the years 2014–2015 from the main districts of Catania, a metropolitan area located in southern Italy. Potential participants were selected based on sex and 10-year age strata from the lists of the general practitioners’ patients (all Italian citizens are registered to a general practitioner, usually covering the area of living of the citizen). Individuals were included if they were older than 18 years old, not pregnant and without invalidating conditions that may have affected memory. Out of 2405 subjects invited to participate in the study, 2044 accepted to participate in the study. For the purposes of the present study, analyses were restricted to participants aged over 65 years, leaving a final sample of 883 individuals included in the present analyses. All study procedures were conducted in accordance with the ethical principles outlined in the World Medical Association’s Declaration of Helsinki (1989). The study protocol has been revised and authorized by the relevant ethical committee.

### 2.2. Background Data

The background information collected for each participant included sex, age at the time of recruitment, highest level of education attained, current occupational status (or, in the case of retired individuals, the main occupation held during their working life), level of physical activity, and smoking habits. Educational attainment was classified into three categories: (i) low (primary or secondary school), (ii) medium (high school), and (iii) high (university degree). The level of physical activity was assessed through the International Physical Activity Questionnaire (IPAQ) and subsequently grouped into three categories: (i) low, (ii) moderate, and (iii) high [45]. Smoking behavior was classified as (i) never smoker, (ii) former smoker, or (iii) current smoker.

### 2.3. Dietary Assessment

Dietary intake was evaluated using two versions of food frequency questionnaires (FFQs), a long and a short form, both previously validated in the Sicilian population [46,47]. The consumption of seasonal foods was recorded for the period in which they were available and subsequently adjusted to reflect their proportional annual intake. Based on the reported food consumption, the energy content as well as macro- and micronutrient intake were calculated using the food composition tables provided by the Council for Research in Agriculture and Analysis of Agricultural Economy (CREA) [48]. For each participant, individual food consumption (expressed in milliliters or grams) was estimated according to standard portion sizes and converted to daily intake. These values were then matched with the databases to obtain average energy content and nutrient composition per 100 mL or grams of each food item. Finally, daily energy, nutrient, and polyphenol intakes were calculated by multiplying the content of each component by the corresponding daily consumption.

### 2.4. Adherence to the Mediterranean Diet

Adherence to the Mediterranean diet was evaluated using a score derived from the literature [49]. The scoring system assigned 2 points to the highest consumption category, 1 point to the intermediate category, and 0 points to the lowest category for food groups characteristic of the Mediterranean diet, including fruits, vegetables, legumes, cereals, fish, and olive oil. Conversely, for food groups not typical of the Mediterranean diet, such as meat and dairy products, 2 points were assigned to the lowest consumption category, 1 point to the intermediate, and 0 points to the highest. Moderate alcohol consumption was considered optimal and contributed positively to the adherence score. The overall score incorporated nine food categories, ranging from 0 points (indicating lowest adherence) to 18 points (indicating highest adherence) [50]. For this study, the upper quartile of the overall score was deemed as having high adherence to the Mediterranean diet.

### 2.5. Mental and Cognitive Health Status

To assess a potential association between mental and cognitive health status with loneliness and aloneness, a subgroup analysis was conducted, taking into account potential variables of interest. Participants’ sleep quality was evaluated using the Pittsburgh sleep quality index (PSQI) [51], also validated for the Italian population [52]. The questionnaire included 19 items clustered into seven domains (sleep quality, sleep latency, sleep duration, habitual sleep efficiency, sleep disturbance, use of sleeping medications, and daytime dysfunction) with answers categorized on a four-point scale ranging from 0 (better) to 3 (worse). The total score was obtained by calculating the sum of the seven domain scores, ranging from 0 to 21 points, with a higher score indicating worse sleep quality. A result of <5 on global PSQI score is indicative of adequate sleep quality.

To test the reporting of depressive symptoms, the Center for the Epidemiological Studies of Depression Short Form (CES-D-20) [53], also validated for the Italian population [54], has been administered. Briefly, CES-D consists of 20 items commonly used to screen for depressive symptoms in the general population. Each item of the scale rates the frequency of each mood or symptom ‘during the past week’ on a 4-point scale ranging from 0 (rarely or none of the time [less than 1 day]) to 3 (most or all of the time [5–7 days]). A score is assigned by totaling all items (after reversing the positive mood items); total scores can range from 0 to 30, with higher scores suggesting greater severity of symptoms, and a score ≥ 16 indicating having depressive symptoms [55,56].

Reporting of stress symptoms was assessed through the application of the Perceived Stress Scale, based on a 14-item questionnaire also validated in the Italian setting [57] with answer options ranging from 0 (never) to 4 (always) and the total score ranging from 0 (minimum) to 56 (maximum). The sex-specific median value was considered as cutoff point to define high or low perceived stress.

Cognitive health was evaluated using the Short Portable Mental Status Questionnaire (SPMSQ) [58], a 10-item screening tool also used in the Italian population [59]. The pre-defined categories for interpretation of the screening tool were (i) intact status, less than 3 errors; (ii) mild impaired, 3 to 4 errors; (iii) moderate impaired, 5 to 7 errors; and (iv) severe impaired status, 8 or more errors. For this study, we considered severe impaired status as a cut-off point for impaired cognitive health.

### 2.6. Participants’ Loneliness and Aloneness

Participants’ subjective feelings of loneliness and objective social integration were evaluated using direct questions as well as items derived from established instruments designed to assess depressive symptoms and quality of life. Feelings of loneliness were assessed using specific items from validated questionnaires (such as the CES-D-20), including statements like ‘I felt lonely.’ A frequency threshold of three times per week was applied to categorize participants as experiencing low or high levels of loneliness. Aloneness was assessed through direct questions that inquired whether participants were part of any social networks, such as family, friends and neighbors, or religious communities, in order to measure aloneness. The median number of social interactions was used as a cut-off to classify participants into low or high aloneness. 

### 2.7. Statistical Analysis

High adherence to the Mediterranean diet was defined as the primary outcome of interest. Categorical variables are presented as absolute numbers and percentages, while continuous variables are reported as means with standard deviations. Group differences for categorical and continuous variables were assessed using the Chi-square test and ANOVA, respectively, or the Kruskal–Wallis test for non-normally distributed variables. Logistic regression analyses were conducted to calculate the odds ratios (ORs) and 95% confidence intervals (CIs) for the association between mental and cognitive health status and the outcomes of interest. Also, logistic regression analyses were used to test the association between high adherence to the Mediterranean diet and the variety of outcomes investigated. The associations were calculated based on fitted unadjusted (Model 1) and multivariable logistic regression models adjusted for age, sex, marital status, educational status, physical activity level, BMI, and smoking status (Model 2). A significance level of 0.05 was applied, and all *p*-values reported are two-sided. Statistical analyses were performed using SPSS version 29 (SPSS Inc., Chicago, IL, USA).

## 3. Results

The median number of social activities among participants was two per week, with 330 individuals (37.4%) classified as more socially isolated and 531 (60.1%) engaging in more than two activities per week. Regarding loneliness, 207 participants (23.4%) reported feeling lonely more than three times per week. The background characteristics of the participants in relation to loneliness and social isolation are presented in Table 1. Perception of loneliness was more common among women and non-smokers than their counterparts. No other background variables were found related to loneliness and aloneness.

Table 2 presents the distribution of nutrients and major food groups in relation to perceived loneliness and aloneness. Regarding food groups, cereal consumption was slightly higher among individuals who did not feel lonely or socially isolated, whereas it tended to decrease in groups reporting loneliness or aloneness. An opposite trend was observed for alcohol, with higher consumption among those who engaged less frequently in social activities. Concerning macronutrients, individuals who felt lonelier reported a lower intake of carbohydrates and fiber, along with an overall lower energy intake.

Table 3 shows the frequency distribution of mental and cognitive health status with the variables of interest of the study. Interestingly, impaired cognitive status, depressive symptoms, and perceived stress (but not sleep quality) were strikingly observed in individuals reporting feeling loneliness but not with actual social isolation (Table 3).

A total of 134 (15.2%) participants fell into the highest quartile of the MEDI-LITE score and were therefore considered to have high adherence to the Mediterranean diet. Table 4 presents the association between loneliness, aloneness, and high adherence to the Mediterranean diet. Both the unadjusted and adjusted models indicated that participants who reported feelings of loneliness and aloneness were less likely to adhere to the Mediterranean diet. After adjusting for potential confounding factors, both loneliness and aloneness were inversely associated with higher adherence to the Mediterranean diet (OR = 0.28, 95% CI: 0.15, 0.51, and OR = 0.26, 95% CI: 0.12, 0.54, respectively). Notably, individuals with higher levels of social engagement showed greater adherence to the diet, with adherence increasing in relation to the number of activities carried out within family and church contexts, but inversely with friends.

## 4. Discussion

The findings from the present study showed that social isolation and loneliness may be associated with dietary habits, indicating a potential reciprocal influence between these factors. In particular, the results highlighted that participants who reported feelings of loneliness exhibited lower adherence to the Mediterranean diet, a dietary model recognized as a paradigm of balanced and healthy nutritional pattern in the same population examined in this study [60]. The results of this study are supported by a rationale presented in the scientific literature. A systematic review indicates that nearly all studies examined report a relationship between loneliness or social isolation and eating behaviors generally considered harmful, such as low fruit and vegetable intake and poorer diet quality, with qualitative research further confirming the negative impact of loneliness and social isolation on dietary habits [23]. Among individual studies of relevance, an investigation conducted in the United States evaluated the dietary adequacy of elderly individuals to determine whether factors such as loneliness, social isolation, or physical health were related to nutrient intake, revealing that loneliness was associated with dietary inadequacies [61]. Concerning the Mediterranean area, a study conducted in Spain on older adults during the COVID-19 lockdown showed that higher loneliness scores were associated with a decline in diet quality [62]. Several hypothetical models have been developed to explain the mechanisms through which psychosocial factors may interact and influence diet quality. Previous studies have highlighted the importance of so-called “life transition points,” critical moments characterized by significant changes in daily routines and social relationships (i.e., living alone or losing a partner) which may directly impact food choices by altering both the availability of time and resources for meal preparation and the social support and established habits related to eating, potentially affecting overall diet quality [63,64]. Retirement has been associated with a deterioration in overall diet quality with an increase in the intake of saturated fats and alcohol and a decrease in the consumption of fish and complex carbohydrates leading to a reduced adherence to healthy dietary patterns such as the Mediterranean diet [65]. Many studies have highlighted this trend: cross-sectional surveys on health, aging, and retirement conducted in the Czech Republic, Poland, and Hungary demonstrated that social isolation was associated with a lower likelihood of daily fruit and vegetable intake in the Czech Republic and Poland, while no significant association was observed in Hungary [66]. Similarly living alone and eating meals in isolation is associated with lower dietary variety and a reduced nutritional adequacy score with decreased consumption of fruit vegetables and fish and a greater tendency toward less balanced dietary patterns [67]. These findings can be explained by the fact that the reorganization of daily routines involves a greater reliance on quick or processed meals and a lower motivation to maintain healthy eating practices in the absence of mealtime companions, while distressing transitions such as separation or widowhood may increase the use of food-related coping behaviors with a preference for high-calorie comfort foods and irregular eating patterns [68,69]. Furthermore, these challenges are compounded by the physiological reduction in appetite that often occurs with aging, which may further limit adequate nutrient intake and hinder the ability to maintain a balanced and healthy diet in older adults [70]. Finally, older individuals may underestimate the importance of diet quality as well as their capacity to recognize it [71,72]. Other evidence suggests that diet itself may strengthen resilience and individuals’ capacity to cope with psychological challenges [73]. In particular, several studies have shown that healthy dietary patterns, such as the Mediterranean diet, are associated with greater psychological resilience and a reduction in depressive symptoms [74]. It is hypothesized that dietary components of these patterns, such as omega-3 fatty acids, polyphenols, and fiber, may play a role in brain health by reducing neuroinflammation, improving cognitive function, and modulating mood, resulting in positive effects on diet quality and psychological well-being [75]. Conversely, high consumption of ultra-processed foods, rich in refined sugars, saturated fats, and artificial additives, has been linked to negative mental health outcomes, including depressive and anxiety symptoms [76]. The mechanisms underlying these effects include systemic inflammation, alterations in gut microbiota, and metabolic dysfunction, which may impair brain function and mood [77]. Furthermore, the high caloric content and low nutritional density of these foods can increase the risk of obesity and other chronic conditions, further exacerbating mental health outcomes [78].

In addition, advancing age can contribute to a decline in overall health and to physical difficulties related to eating, such as problems with chewing or meal preparation, which compound the effects of previously mentioned psychosocial factors, thereby influencing the variety and overall quality of the diet [79]. Among the various mechanisms potentially involved, participation in social and recreational activities has been associated with greater psychological resilience and enhances a sense of purpose and belonging, promoting more regular and mindful eating behaviors [80]. These findings suggest that, although aging inevitably involves physical and social changes, promoting active social networks and participation in collective activities may mitigate the negative effects of loneliness on eating habits, supporting a more varied and nutritionally balanced diet [81]. They also underscore the need for greater awareness and understanding of the interactions between these complex aspects of health in order to guide the development of targeted interventions. 

Consistent with the findings that highlighted the association between feelings of loneliness and lower adherence to the Mediterranean diet, individuals with higher levels of social integration and participation showed greater adherence to this dietary pattern, with a progressive increase proportional to the number of activities carried out within family and religious contexts [82]. There is a paradigm linking food consumption not only to maintaining health but also to the hedonic pleasure associated with eating [72]. A reduction in personal enjoyment of eating or participation in social activities related to meal preparation and consumption, such as sharing meals with family members, relatives, or friends, may contribute to an overall decline in diet quality, including its nutritional value [83,84]. Individuals who live alone may be less motivated to cook for themselves, potentially developing “food apathy”, defined as a diminished attention or interest in food [85]. This disengagement from eating can lead to diets that are less varied and nutrient-dense, with potential long-term health implications [86]. In contrast, individuals who feel lonely but still live with family members may continue to prepare meals due to the physical presence of others, yet they may preferentially choose comfort foods or items of lower nutritional quality, influenced by negative mood states or emotional distress [87].

Interestingly, feeling loneliness rather than actual aloneness was related to mental and cognitive health conditions in this study population. Such an association, together with the level of diet quality (assessed through the Mediterranean diet adherence), may have biological meaning. The Mediterranean diet, characterized by high consumption of fruits, vegetables, legumes, whole grains, fish, and olive oil, and a moderate intake of wine, has been consistently associated with lower risk of neurodegenerative and affective disorders [88,89,90]. Its beneficial effects on brain health are thought to derive from the synergistic action of multiple nutrients and bioactive compounds that influence neuroinflammatory pathways, oxidative stress, and vascular integrity [91,92]. Adherence to the Mediterranean diet has been linked to reduced systemic inflammation and improved endothelial function, both of which play crucial roles in maintaining cerebral perfusion and neuronal integrity [93,94]. Diets rich in polyphenols, (poly)phenols and antioxidants may attenuate neuroinflammatory cascades by downregulating pro-inflammatory cytokines (such as IL-6 and TNF-α) and enhancing the anti-inflammatory defense systems [95,96]. Also, a potential involvement of the gut microbiota and its regulation via better diet quality has been hypothesized to play a role through a variety of mechanisms leading to reduced neuroinflammation [97,98]: the Mediterranean diet has been in fact related to positive modulatory effect on intestinal microbiome, favoring bacterial taxa involved in the synthesis of several bioactive compounds, such as short-chain fatty acids, that may exert promoting health effects [99,100] These mechanisms could contribute to the preservation of cognitive performance and reduce the vulnerability to depressive symptoms and perceived stress observed in individuals with higher diet quality.

From a neurobiological perspective, nutritional patterns consistent with the Mediterranean diet may support neuroplasticity and neurotransmitter synthesis through the modulation of brain-derived neurotrophic factor (BDNF) and monoaminergic systems [101]. Conversely, loneliness has been independently associated with dysregulated hypothalamic–pituitary–adrenal (HPA) axis activity, elevated cortisol levels, and chronic low-grade [102] inflammation, which are detrimental to both mood regulation and cognitive processes [102,103,104]. Therefore, individuals with lower Mediterranean diet adherence might experience a cumulative biological burden through the convergence of poor diet and psychosocial stress, amplifying the risk of depressive symptoms and cognitive impairment. In contrast, higher adherence to the Mediterranean dietary pattern could act as a protective buffer, mitigating the adverse physiological consequences of loneliness through anti-inflammatory and neuroprotective mechanisms.

This study has some limitations that warrant attention when considering the reported findings. First, its cross-sectional design only permits the identification of associations between variables, preventing any conclusions about causal relationships. Nevertheless, the findings offer valuable insights into a cluster of interrelated variables that should be examined collectively, rather than in isolation. Second, the tools used to assess dietary intakes may suffer from recall bias, potentially leading to over- or underestimation of true dietary intakes. Third, the outcomes investigated in this study have been evaluated through simple questions and may not fully describe the multiplicity of complex psychosocial conditions. Finally, although several variables have been taken into account as potential confounding factors, other unmeasured factors (such as socioeconomic status beyond education or other chronic diseases) may still affect the results with residual confounding.

## 5. Conclusions

In conclusion, the association between loneliness, aloneness, and poor diet quality is an important area of research with important public health implications. Future research is needed to better understand the mechanisms underlying this association and to develop effective interventions to promote healthy eating in individuals who are lonely or alone. The results from this study ultimately emphasise the importance of targeted interventions aimed at addressing these issues.

## Figures and Tables

**Table 1:** Background characteristics by loneliness and aloneness.

	Loneliness		*p*-Value	Aloneness		*p*-Value
	No	Yes		No	Yes	
**Age (y), mean (SD)**	65.3 (9.6)	63.8 (9.3)	0.053	64.6 (9.4)	65.3 (9.8)	0.302
**Sex, n (%)**			0.003			0.127
Male	310 (81.2)	72 (18.8)		221 (57.9)	161 (42.1)	
Female	366 (73.1)	135 (26.9)		310 (61.9)	191 (38.1)	
**Marital status, n (%)**			0.131			0.161
Single/divorced/widowed	193 (71.0)	79 (29.0)		163 (59.9)	109 (40.1)	
Married/cohabitant	483 (79.1)	128 (20.9)		368 (60.2)	243 (39.8)	
**Education level, n (%)**			0.271			0.446
Low	349 (77.4)	102 (22.6)		263 (58.3)	188 (41.7)	
Medium	222 (77.9)	63 (22.1)		174 (61.1)	111 (38.9)	
High	105 (71.4)	42 (28.6)		94 (63.9)	53 (36.1)	
**Smoking status, n (%)**			0.002			0.992
Non-smoker	365 (73.4)	132 (26.6)		299 (60.2)	198 (39.8)	
Current	140 (74.9)	47 (25.1)		113 (60.4)	74 (39.6)	
Former smoker	171 (85.9)	28 (14.1)		119 (59.8)	80 (40.2)	
**BMI, n (%)**			0.894			0.835
Normal	225 (75.0)	75 (25.0)		182 (60.7.)	118 (39.3)	
Overweight	259 (76.4)	80 (23.6)		199 (58.7)	140 (41.3)	
Obese	147 (76.6)	45 (23.4)		117 (60.9)	75 (39.1)	
**Physical Activity score**			0.735			0.346
Low	140 (71.4)	56 (28.6)		119 (60.7)	77 (39.3)	
Medium	274 (74.1)	96 (25.9)		219 (59.2)	151 (40.8)	
High	131 (71.6)	52 (28.4)		120 (65.6)	63 (34.4)	

**Table 2:** Major food groups and nutrient distribution by loneliness and aloneness.

	Loneliness		*p*-Value	Aloneness		*p*-Value
Mean (SD)	No	Yes		No	Yes	
Cereals (g/d)	238.1 (135.9)	193.7 (110.9)	<0.001	236.6 (136.8)	214.2 (122.8)	0.013
Fish (g/d)	65.9 (54.7)	64.1 (83.3)	0.717	63.8 (59.3)	68.0 (67.1)	0.330
Total meat (g/d)	70.9 (40.6)	63.3 (34.1)	0.015	69.4 (40.4)	68.7 (37.7)	0.806
Red meat (g/d)	34.2 (27.3)	29.3 (21.5)	0.018	33.7 (27.5)	32.1 (24)	0.349
Red processed meat (g/d)	12.7 (14.7)	16 (22.6)	0.013	13.8 (16.6)	13.1 (17.4)	0.584
Eggs (n eggs)	2.7 (5.1)	2.6 (4.7)	0.840	2.5 (4.5)	3.0 (5.7)	0.188
Red wine (glass)	45.7 (85.7)	50 (93.9)	0.534	32.5 (64)	68.1 (111.1)	<0.001
White wine (glass)	13.1 (40.2)	16.5 (48.4)	0.301	10.8 (29.5)	18.5 (56)	0.008
Dairy (g/d)	181.5 (171.1)	220.9 (173)	0.004	187.6 (171.1)	195.5 (174.2)	0.503
Olive oil (n spoons)	7.4 (3.1)	7.0 (3.2)	0.106	7.4 (3.1)	7.2 (3.2)	0.283
Fruit (g/d)	412.7 (313.3)	391.8 (327.6)	0.406	415.3 (322.2)	396.4 (308.2)	0.386
Vegetables (g/d)	256.3 (135.5)	278 (171.8)	0.059	263.7 (138.1)	257.8 (155)	0.554
Legumes (g/d)	38.4 (35.3)	34.9 (38.1)	0.223	37.5 (35.3)	37.7 (37.1)	0.949
Nuts (g/d)	22.1 (34.9)	16.2 (19)	0.021	18.6 (29)	23.8 (35.9)	0.018
Protein (g)	85.8 (26.5)	81.4 (35.1)	0.052	85.1 (28.3)	84.2 (29.5)	0.640
Fats (g)	60.2 (20.3)	56.9 (26.7)	0.062	58.8 (21.2)	60.4 (23.2)	0.313
Cholesterol (mg)	191.4 (76.1)	178.6 (114.5)	0.064	186.9 (86.1)	190.7 (87.7)	0.520
Carbohydrates (g)	310 (113.2)	270.5 (92.3)	<0.001	303.6 (111.7)	296.5 (107.1)	0.348
Total fiber (g)	33.4 (13.9)	30.6 (13.7)	0.010	33 (13.6)	32.3 (14.3)	0.462
Energy (kcal)	2091.5 (642.3)	1904.8 (663.6)	<0.001	2040.1 (648.1)	2059.1 (658.2)	0.672
Energy (kJ)	8473.4 (2647.3)	7719.4 (2715.6)	<0.001	8271.3 (2672.7)	8334.8 (2696.8)	0.730
Sodium (mg)	2760.6 (1073.1)	2759.3 (1132.4)	0.988	2777.6 (1072.7)	2734.3 (1108.4)	0.562
Potassium (mg)	3699.2 (1271.7)	3648.3 (1580.1)	0.635	3688.5 (1309.7)	3685.5 (1409.4)	0.975
Alcohol (g)	8.0 (12.2)	9.4 (14.3)	0.171	6.0 (9.7)	11.7 (15.6)	<0.001
Vitamin A retinol eq. (µg)	864.4 (402.1)	891.3 (487.3)	0.424	879.7 (422.2)	857.2 (425.7)	0.439
Vitamin C (mg)	159.5 (95.5)	159.3 (101.5)	0.987	162.2 (99)	155.2 (93.6)	0.292
Vitamin E (mg)	8.7 (3)	8.6 (4)	0.841	8.6 (3.1)	8.7 (3.5)	0.567
Saturated fats (%)	24.0 (9.1)	22.1 (10.5)	0.015	23.3 (9.6)	23.8 (9.4)	0.485
Mono-unsaturated fats (%)	25.7 (8.1)	24 (10.8)	0.017	25.1 (8.3)	25.6 (9.6)	0.394
Poly-unsaturated fats (%)	11.2 (4.2)	10.2 (4.9)	0.003	11 (4.3)	11.0 (4.6)	0.873
Vitamin d (µg)	5.7 (4.8)	5.6 (7.5)	0.961	5.5 (5.4)	5.9 (5.7)	0.282
Vitamin B12 (µg)	6.1 (3.5)	6.4 (6.3)	0.286	6.1 (4.3)	6.3 (4.3)	0.507
Total omega-3 (g/d)	1.8 (0.8)	1.6 (1.1)	0.015	1.8 (0.8)	1.8 (0.9)	0.831

**Table 3:** Association between mental and cognitive health with loneliness and aloneness.

	Loneliness		*p*-Value	Aloneness		*p*-Value
	No	Yes		No	Yes	
**Impaired cognitive status, n (%)**	20 (3.0)	62 (30.0)	<0.001	50 (9.4)	32 (9.1)	0.485
**Depressive symptoms, n (%)**	12 (1.8)	200 (96.6)	<0.001	122 (23.0)	90 (25.6)	0.211
**Perceived stress, n (%)**	248 (36.7)	185 (89.4)	<0.001	262 (49.3)	171 (48.6)	0.439
**Inadequate sleep quality, n (%)**	461 (68.2)	142 (68.6)	0.493	359 (67.6)	244 (69.3)	0.323

**Table 4:** Association between loneliness and aloneness with high adherence to the Mediterranean diet.

	High Adherence to the Mediterranean Diet, OR (95% CI)	
	Unadjusted	Adjusted *
**Loneliness (feeling alone)**	0.28 (0.15, 0.51)	0.26 (0.12, 0.54)
**Aloneness (being alone)**	0.44 (0.29, 0.67)	0.44 (0.26, 0.75)
No. family activities	2.23 (1.78, 2.78)	2.78 (1.98, 3.20)
No. friends activities	1.18 (0.96, 1.45)	0.70 (0.52, 0.96)
No. church activities	1.96 (1.20, 3.18)	4.17 (2.15, 7.87)

* Model adjusted for age, sex, marital status, educational status, physical activity level, BMI, and smoking status.

## Data Availability

The data presented in this study are available on request from the corresponding author due to confidentiality regulation.

## Abbreviations

The following abbreviations are used in this manuscript:

ANOVA	Analysis of Variance
BDNF	Brain-Derived Neurotrophic Factor
BMI	Body Mass Index
CES-D-20	Center for Epidemiological Studies Depression Scale
CI	Confidence Interval
CREA	Council for Research in Agriculture and Analysis of Agricultural Economy
FFQ	Food Frequency Questionnaire
HPA axis	Hypothalamic–Pituitary–Adrenal axis
IL-6	Interleukin-6
IPAQ	International Physical Activity Questionnaire
OR	Odds Ratio
PSQI	Pittsburgh Sleep Quality Index
SPMSQ	Short Portable Mental Status Questionnaire
TNF-α	Tumor Necrosis Factor alpha
WHO	World Health Organization
